# A Systematic Review of Open, Laparoscopic, and Robotic Inguinal Hernia Repair: Management of Inguinal Hernias in the 21st Century

**DOI:** 10.3390/jcm14030990

**Published:** 2025-02-04

**Authors:** Sergio Huerta, Amanda M. Garza

**Affiliations:** 1 VA North Texas Health Care System, Dallas, TX 75216, USA; 2 University of Texas Southwestern Medical Center, Dallas, TX 75390, USA; amanda.garza@utsouthwestern.edu

**Keywords:** groin hernia, femoral hernia, Shouldice, Lichtenstein

## Abstract

**Background:** In the 21st century, the management of groin hernias (GHs) has evolved from watchful waiting (WW) to robotic hernia repair (RHR). The present study interrogates the status of robotics in the context of current repairs and provides one author’s perspectives. **Methods:** A systematic review was undertaken using Preferred Reporting Items for Systematic Reviews and Meta-Analysis (PRISMA) guidelines for studies comparing open (OHR) to robotic hernia repair (RHR); RHR to laparoscopic hernia repair (LHR); or OHR vs. LHR vs. RHR. The historical context was extracted from previous reviews. **Results:** Fifty-four studies were included in the analysis. Three techniques have withstood the test of time: OHR (tissue and mesh repairs), laparo-endoscopic (TEP and TAPP), and RHR. The literature indicates that RHR is safe and effective for the management of groin hernias. Operative times and costs remain a concern when using this technique. While the number of overall complications with RHR is similar to OHR, in a minority of cases, complications are more consequential with the robotic platform. **Conclusions:** RHR has emerged as an unequivocally powerful technique for the management of GHs. OHR remains the technique of choice for local/regional anesthesia, posterior recurrences, and in centers that lack other platforms. In low- and middle-income countries, OHR is the most utilized technique. Centers of excellence should offer all techniques of repair including WW.

## 1. Introduction

One in four adult men are at risk of developing an inguinal hernia in their lifetime, making inguinal hernia repair one of the most common operations performed by general surgeons worldwide [1]. The incidence of inguinal hernias increases with age, such that the likelihood of a 70-year-old man developing one is up to 50% [2]. Complications also increase with age, especially when repair is performed in an emergency setting [3].

Globally, 20 million inguinal hernia operations are performed annually [4]. In the United States alone, 800,000 of these procedures are performed every year [5]. In the 170 Veteran Administration hospitals across the United States, 11,000 hernias are repaired yearly [6,7]. In France and the United Kingdom, 100,000 and 80,000 inguinal hernia repairs are performed every year, respectively. Two million Chinese individuals are diagnosed with an inguinal hernia annually [8].

**Figure 1:**
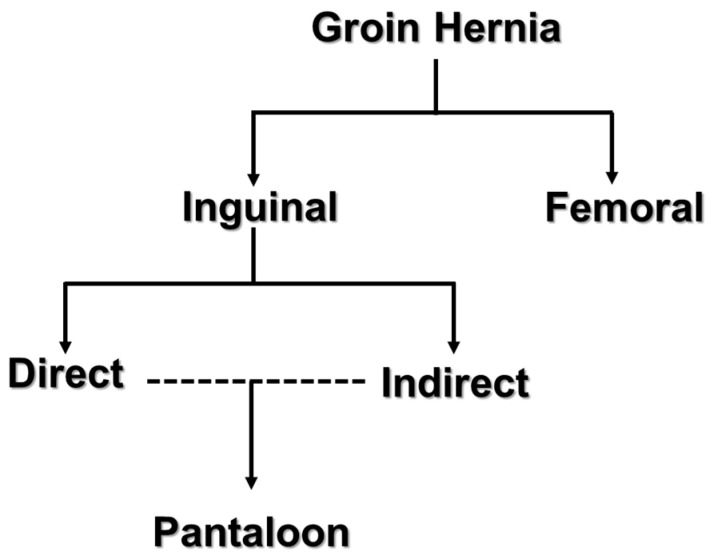
Groin hernia types. Inguinal hernias can be direct or indirect. An indirect and direct inguinal hernia on the same side is called a pantaloon hernia.

Inguinal and femoral hernias fall within the definition of groin hernias, both occurring within the myopectineal orifice (Figure 1). This oval shaped region is bordered superiorly by the conjoint tendon, inferiorly by Copper’s ligament, laterally by the iliopsoas muscle, and medially by the rectus sheath [9].

Femoral hernias are much less common than inguinal hernias and comprise 2–4% of all groin hernias. An analysis of 3980 femoral hernias showed that femoral hernias were more common in women compared to men (63% vs. 37%) and are more likely to present with incarceration compared to inguinal hernias (36% vs. 4.9%) [10].

In 2005, 48 billion dollars of health care costs in the United States were attributed to this operation [11,12]. This procedure, therefore, accumulates substantial health care costs and has important clinical implications, such that every aspect that could potentially lower complications and costs should be analyzed.

**Figure 2:**
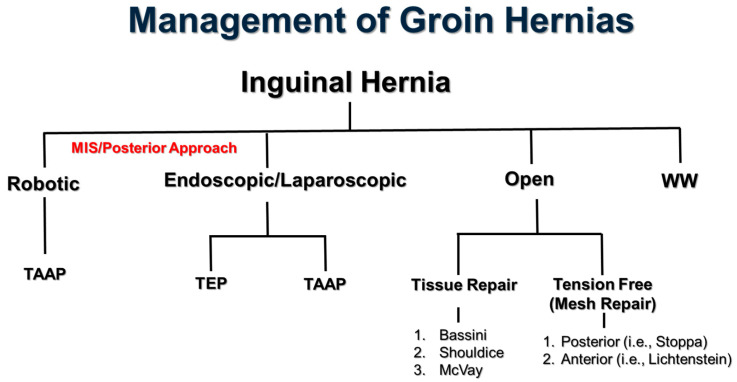
The management of groin hernias has considerably evolved since the 1950s. Minimally invasive techniques (MIS) include robotic and laparo-endoscopic approaches [transabdominal preperitoneal (TAPP) and total extraperitoneal (TEP)]. Watchful waiting (WW) has also emerged as a strong option in the management of inguinal hernias [16]. A technique the combines the tissue repair and tension free repair (without mesh) uses aponeurosis of the external oblique (the Desarda method [23]).

Mortality following elective inguinal herniorrhaphy is unlikely and commonly not reported. However, complications from inguinal hernias ranging from mild urinary retention to severe chronic pain and recurrence can occur in up to 35% of cases [13]. Unfortunately, outcome reporting for inguinal hernia repair has not been standardized [14]. Currently, the most important long-term complications of inguinal hernia repair are recurrence (~1.0–5.0%) and chronic inguinal pain (~10%) [15]. Prior to the 1970s, the only approach to inguinal hernia repair was via tissue techniques. Since then, the management of groin hernias has rapidly evolved on both sides of the spectrum, from watchful waiting (WW) [16] to robotic techniques [17] (Figure 2).

Before tension-free repair emerged as the major form of management for inguinal hernias in the 1970s [18,19], tissue repairs were the only techniques able to address inguinal hernias [20]. Easy reproducibility and a markedly decreased rate of recurrence of the tension-free repair has made it the dominant strategy in the United States, as well as low- and middle-income countries (LMICs) [21,22].

In 1990, Ger described a laparoscopic ligation of the sac in animals [24]. Soon after, multiple laparoscopic techniques were introduced; the most popular of which were the laparoscopic trans-abdominal preperitoneal (TAPP) approach and the total extraperitoneal approach (TEP) [25].

Urologists popularized the robotic platform for prostatectomies, and with this they identified inguinal hernias that needed to be repaired [26]. Most commonly, the robotic approach to inguinal hernias is an extension of the laparoscopic TAPP repair approach. With new techniques, new questions rapidly emerged [27].

Level I evidence comparing open to laparoscopic inguinal hernia repair is currently limited in the literature [28]. To date, there is no head-to-head comparison of the open (OHR), laparoscopic/endoscopic (LHR), and robotic (RHR) hernia repairs in randomized controlled trials. However, single-institution studies comparing all three techniques [17,29,30] as well as other studies investigating the open compared to the robotic approach are available in the literature [31,32,33,34,35,36].

The present report is a comprehensive review of the status of RHR as compared to OHR, LHR, or both. The aim of this review is to assess the role of the robotic approach to inguinal hernias by comparing it to other methods within a historical perspective. The manuscript concludes with a current personal perspective from the authors.

## 2. Materials and Methods

This systematic review was conducted following the Preferred Reporting Items for Systematic Reviews and Meta-Analysis (PRISMA) guidelines [37]. The lead author reviewed the articles, and a single screener decided which articles to include or exclude, while discrepancies or indecisions were resolved via discussion among the authors. Individual study bias was mitigated by reviewing and confirming the appropriate sources indicated. The bibliographies of relevant studies were hand-searched to identify any additional studies. All duplicate articles, abstracts, review articles, and letters to the editor were excluded. The last day of the search was December 15, 2024. Various combinations of keywords including “hernia”, “inguinal hernia”, “femoral hernia”, “groin”, “laparoscopy”, “endoscopy”, “robotic”, “laparo-endoscopic”, and “minimally invasive” were used for our searches. No time restrictions (beyond that of the existing databases) or language restrictions were imposed. Databases including PubMed, MEDLINE (via PubMed), and Embase were initially searched. Subsequently, Cochrane Library, Google, Google Scholar, and ResearchGate were utilized to search for and acquire reports that were new and/or unavailable from the previous databases. Further manuscripts were identified by close examination of the references of the index papers and main reviews on this subject. These manuscripts were included in our review if they were appropriate references and did not duplicate our original findings reviewed elsewhere.

**Figure 3:**
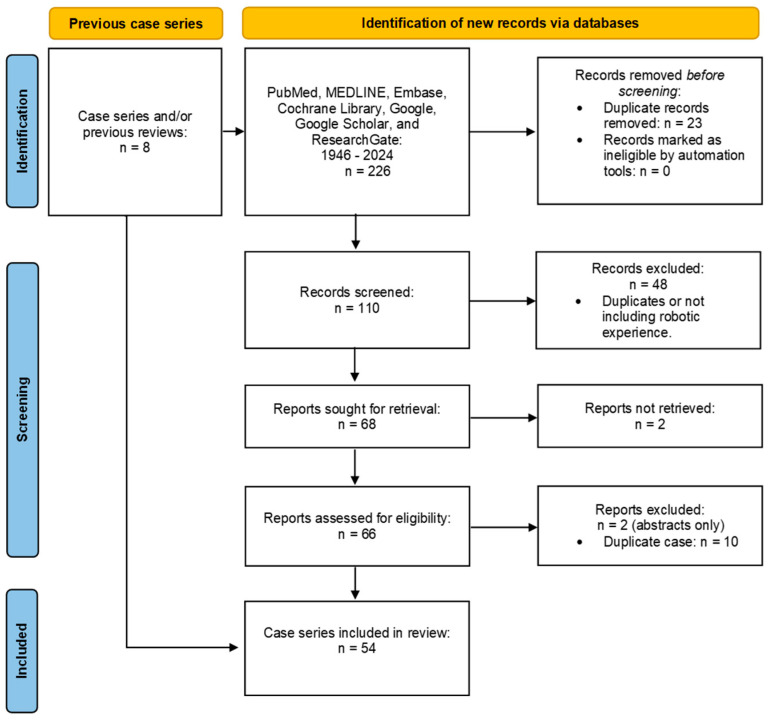
PRIMA flow-chart describing the process of our literature search.

The PRISMA flow-chart depicts the screening process (Figure 3). All of the abstracts were analyzed within an EndNote group to eliminate irrelevant and duplicated studies. Google translate was utilized to translate articles in other languages to English. Full text for a handful of articles was unavailable for a variety of reasons. These include a lack of electronic copies, restrictions by foreign countries, incomplete scanning, and older manuscript date.

Because OHR vs. LHR has been previously reported extensively elsewhere [38,39,40,41,42,43,44,45,46,47,48], these analyses were not included in the present report, but the historical perspective within those manuscripts was reviewed by the primary author (SH). All outcomes reported in the results section show how the RHR compares to either LHR, OHR, or both.

## 3. Results

### 3.1. Literature Review

In total, 54 studies were included in this systematic review. In general, smaller studies provided more granular data (i.e., the rate of specific complications such as recurrence and inguinodynia). Larger studies provided general trends. There was a great deal of variability in the data reported in these studies, including documenting outcomes, comparisons, study types, and bias, especially those exclusively addressing the robotic technique.

#### 3.1.1. Study Type

Most manuscripts reported retrospective, single-surgeon, single-center practice robotic experience [17,32,33,36,48,49,50,51,52,53,54,55,56,57,58,59,60,61,62,63,64,65,66,67,68,69,70,71,72,73]. Ten studies reported multicenter experience [34,35,74,75,76,77,78,79,80,81]. Nine papers interrogated large databases of analyses. These included the American College of Surgeons National Quality Surgery Improvement Program Database (ACS NSQIP) [30], Veteran Affairs Surgery Quality Improvement Project (VASQIP) [7], The Healthcare Cost and Utilization Project-State Inpatient Databases and the American Hospital Association Annual Health Survey Databases [82], The Vizient clinical database [83], The Florida Agency for Health Care Administration database [84], The Abdominal Core Health Quality Collaborative Registry [85], The New York Statewide Planning and Research Cooperative System (SPARCS) administrative database [86], The Abdominal Core Health Quality Collaborative database [87], and the Premier hospital database [88].

#### 3.1.2. Comparisons of Techniques

This study included 10 manuscripts that described either single-surgeon or institutional experience with RHR [53,57,60,64,65,69,76,80,89,90]. Most studies (n = 27) compared the LHR and RHR techniques [32,36,48,49,51,52,54,58,59,62,63,68,71,72,73,74,75,78,79,81,88,91,92,93,94,95]. Only three studies compared OHR vs. RHR [33,34,35]. Sixteen studies compared all three techniques [7,17,30,50,56,66,70,77,82,83,84,85,86,87,96] (Table 1). There were only two RCTs that exclusively compared LHR vs. RHR [78,92]. There were no randomized controlled trials comparing all three techniques.

#### 3.1.3. The Data from the Studies

The first documented feasibility study for RHR was reported in 2008 [65]. Waite et al. published one of the initial comparative studies between LHR and RHR [72]. There were two randomized controlled trials (RCTs) [78,92] and six systematic reviews [93,94,95,96,97]. Two of the systematic reviews included meta-analyses [91,96]. Some studies focused exclusively on the repair of bilateral inguinal hernias comparing RHR to other platforms [57,62,79]. One study included only obese patients in a comparison between OHR and RHR [35].

#### 3.1.4. Main Findings of Studies

##### Feasibility of RHR

Six studies addressing RHR focused primarily on the feasibility of this approach [32,61,64,65,69,71]. The uniform findings of these descriptive papers were to indicate that the robotic platform was safe and effective for the management of inguinal hernias.

##### Postoperative Pain

Postoperative pain was reported as the only focus of seventeen papers. Postoperative pain was reportedly improved in eight cases using the robotic platform compared to others [34,41,48,53,58,62,72,83]. A similar number of studies (n = 8) showed no difference to other techniques in terms of postoperative pain [50,60,68,70,78,79,81,95]. Only one study showed the robotic platform to be inferior in terms of postoperative pain compared to the others [87] (Table 1).

##### Conversion Rate

Four studies documented similar conversion rates. In two studies the conversion rate was similar in LHR and RHR [74,80] and two showed the robotic platform to be superior to the laparo-endoscopic technique [66,77].

##### Hospital Length of Stay

Hospital length of stay (LOS) appeared in eleven documents. The LOS was similar in six studies [58,62,68,74,91,95], shorter with the robot in four [34,66,82,83], and longer in one [86] (Table 1).

##### Operative Room Times

Operative room (OR) times were the major issue for many studies (n = 27). Most studies (n = 20) reported unequivocally longer OR times with the robotic platform compared to the open and laparo-endoscopic approaches, or both [8,17,30,49,52,54,62,63,68,72,73,74,75,79,91,93,94,96,98,99]. Five studies documented initially longer OR times with the robotic platform, but an improvement after the learning curve had been overcome [33,55,59,60,77]. Two studies reported similar OR times with the robot compared to other techniques [36,58] (Table 1).

##### Cost

In fifteen studies, cost was either the primary focus of the analysis or specifically mentioned as an examined variable. All fifteen studies uniformly showed that costs were higher with the robot compared to other platforms [32,49,50,52,62,63,72,74,82,83,84,91,94]. One study indicated that the cost of the robotic platform for inguinal hernias increased (rather than an expected decrease) overtime [84] (Table 1).

##### Overall Outcomes

Reported overall outcomes ranged from estimated blood loss, seroma and hematoma formation to bowel and vascular complications. Overall outcomes were reported in forty manuscripts. Most studies (n = 28) reported similar outcomes compared to the LHR, OHR, or both [30,33,36,41,48,50,52,53,54,55,57,58,59,60,63,68,73,75,78,79,80,87,91,92,94,95,96,97]. Four studies reported worse overall outcomes compared to the open, laparoscopic, or both techniques [17,49,85,93]. Six manuscripts documented superior overall outcomes with the robotic platform compared to the others [7,32,34,35,62,81,83,86].

Five papers included a specific percentage of overall complications [17,80,93,97,100], including seroma formation, hematoma, and urinary retention. Collectively, the average complications for OHR, LHR, and RHR were 11.2%, 34.0%, and 38.0%, respectively.

Recurrence rate was specifically reported in 10 manuscripts [27,58,63,68,73,75,78,81,85,93]. There was no reported difference in recurrence based on approach. The average recurrence for OHR, LHR, and RHR were 1.0%, 2.7%, and 1.5%, respectively.

Inguinodynia was less commonly reported in many studies. The ones that reported it indicated a 5.0%, 6.8%, and 8.8% rate of inguinodynia for the OHR, LHR, and RHR, respectively [17,81] (Table 1).

## 4. Discussion

While over seventy techniques have been described for the management of inguinal hernias over the past two centuries, only three major different platforms have withstood the test of time and are currently the cornerstone of surgical practice around the world. The first one is open repair, either tissue repair (i.e., Shouldice) [101] or the tension-free technique popularized by Lichtenstein [19]. The second is the laparo-endoscopic technique, including both the total extraperitoneal approach (TEP) and trans-abdominal preperitoneal repair (TAAP), which are currently practiced in many centers in the world [98,102,103]. Today, the most recent technique added to the armamentarium of groin hernia repair is the robotic platform, which is prominently an extension of the TAPP approach and the focus of this manuscript [80,90,104].

With regard to the open approach, the history prior to Marcy and Bassini is less relevant to our current practice [105,106,107,108]. In 1871, Marcy described the need to ligate an indirect hernia sac and the importance of reducing the opening of the internal ring [105,106]. Later, Edoardo Bassini reported 8 recurrences in 208 inguinal hernia repairs (4%). The results of this monumental advancement in hernia surgery did not yield reproducible results, even from pupils of Bassini. Modifications of the original technique were shadowed by complications resulting from penetrating the underlying preperitoneal space [108]. Thus, today, the most reproducible approach to an inguinal hernia with a small learning curve is open mesh repair for unilateral inguinal hernias [19].

The Shouldice institute in Canada performs 7500 hernia repairs a year. The rate of recurrent hernias that are repaired at the Shouldice hospital is approximately 10.4%. The repair of inguinal hernias is exclusively carried out via tissue repair and all patients undergo local anesthesia for the operation. The recurrence rate for indirect and direct hernias is 0.13% and 0.31%, respectively [101]. The hospital has been practicing the same technique for 74 years.

Today, tension-free repair has become the most common technique for the management of inguinal hernias around the globe [19]. Its reproducibility and small learning curve provide a technique that is readily available for most surgeons starting practice in general surgery. The rates of recurrence and overall postoperative complications are acceptable, except for chronic postoperative pain (~10%) [15,109,110,111]. It is also the most common approach in LMICs as it allows for the use of local or regional anesthesia [22].

Notably, within the open techniques, the Desarda method might be the only alternative in areas of the world without access to mesh. The Desarda approach as described by Mohan P. Desarda in 2001 [23] involves using a piece of the aponeurosis of the external oblique to address the weakness of the transversalis fascia. Thus, while this technique is a type of tissue repair, it is also tension free. The outcomes and reproducibility of this approach are similar to those of other methods of hernia repair [112].

The 1990s were not only notable for the popularization of tension-free repair, as laparo-endoscopy began to emerge as a viable technique for the repair of inguinal hernias during this time as well. Bilateral and recurrent hernias became the original common indication for this approach. In 1995, Fitzgibbon reported the results of a multicenter trial showing LHR as an effective technique for inguinal hernias [46]. Today, guidelines for hernia repair recommend the laparo-endoscopic approach for primary, unilateral inguinal hernias, citing a reduction in chronic postoperative pain [113]. In the USA, about 28% of primary unilateral IHRs were performed using LHR techniques between 2005 and 2012 [114]. In 2011, 44% of all inguinal hernia repairs in Austria were performed laparo-endoscopically [71].

Robotic surgery was initially introduced by Cadier et al. in 1997, when a robotic cholecystectomy was performed utilizing the da Vinci robotic platform [115]. In 2000, the Food and Drug Administration (FDA) approved the da Vinci System [Intuitive Surgical Inc. (Sunnyvale, California, USA)] [69,115]. As of 2019, one in four hospitals in the United States had acquired a robot, which has led to an exponential increase in the number of robotic operations performed via the da Vinci system [74]. Today, the use of robotics in general surgery has expanded to just about any intraabdominal procedure, including foregut operations, hepatobiliary operations, bariatric operations, colectomies, and inguinal and ventral hernias [17,69,116,117]. Urologists, cardio thoracic surgeons, gynecologists, pediatric surgeons, and general surgeons perform robotic operations [91]. In 2014, of all robotic cases performed globally, 79% of all robotic-assisted surgeries worldwide were performed in the USA, of which 24% were general surgery cases, with inguinal hernia repairs and cholecystectomies as the most common procedures [35]. The RHR is advocated in its ability to perform more complex cases [116,117]. Some have also proposed better outcomes with RHR vs. OHR [33,34].

**Figure 4:**
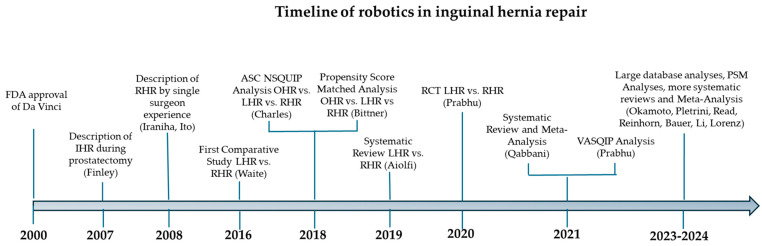
The timeline of the progression of robotics in inguinal hernia repair. IHR = inguinal hernia repair; LHR = laparo-endoscopic hernia repair; RHR = robotic; hernia repair; OHR = open hernia repair; PSM = propensity score matched.

Finley et al. first reported the combined robotic prostatectomy and prosthetic mesh inguinal hernia repair in 2007 [118]. A study of the Premier Healthcare Database ( > 600 hospitals in the US) found that there was an increase in RHR from 0.08% in 2010 (n = 10) to 3.27% in 2015 (n = 625) [88]. In a study by Tsu et al., 26 RHRs were performed for unilateral, primary IHs in 2016. This number tripled in only three years with an associated decrease in both OHR and LHR [63]. In another study at the VA hospital between 2008 and 2019, the rate of robotic inguinal hernias increased from 0.24% to 19.6% [7]. These exponential increases are seen across the USA. Between 2015 and 2017, there was an increase in the number of RHRs from 140,000 to 246,000 in the USA. This was at the expense of a decrease in LHR, but not OHR [119]. However, these numbers still represent only a small fraction of all inguinal hernias repaired in the USA. For instance, from 2010 to 2015, the Premier Healthcare Database identified that of the 102,241 minimally invasive inguinal hernia repairs performed, only 1.1% utilized a robot. Based on the current review and trends, this number is expected to grow significantly (Figure 4).

The present manuscript is the most comprehension review of robotic inguinal hernia repair today, as it compares it to other platforms. The main goal of the present analysis is to determine the status of inguinal hernia repair options in the 21st century with emphasis on the status of the robotic platform. Without a doubt, RHR can be performed with acceptable outcomes compared to LHR and RHR. However, operative room times and costs remain a consistent issue today [49,74,120]. Nonetheless, reports show that operative room times and costs improve as the learning curve progresses [58,68]. RHR allows for more complex cases to be performed, including bilateral and recurrent inguinal hernia repairs. Incidentally found hernias have also been repaired successfully [80]. There is also no doubt that the learning curve for RHR is much smaller than that for LHR [58,68,80].

### 4.1. Perspective

This perspective emanates from a surgeon that has exclusively performed OHRs (~2000) over the past twenty years in a veteran patient population with a high rate of comorbid conditions [3,11,17,121] and in LMICs [22]. I have previously argued that OHR remains the gold standard for IHR [111,121]. While this bias might direct the audience to predict a similar argument in the current management of inguinal hernias, the present review offers an undeniable increase in numbers and popularity of RHR. The use of RHR will continue to increase in centers of excellence. Based on the current trends, I predict that operating room times, as well as costs, will decrease. This will also unquestionably be the result of less experience with the OHR or LHR, as the robotic approach continues to dominate in high-income countries.

Taken together, the main perspective of this author is outlined in the following points:

- In the 21st Century, there should not be a “gold-standard” for the management of inguinal hernias. The approach should be based on the experience of the center, the surgeon, and the patient population (system-based practices). For instance, the following options should be considered.
  - For older patients with substantial co-morbidities and an asymptomatic inguinal hernia, a WW approach might be the standard of care.
  - In most LMICs in the globe, where resources are scarce, the best and most reproducible technique might be tension-free repair under local, regional, or general anesthesia.
  - In countries where mesh and general anesthesia might not be available, the best technique might be the one described by M.P. Desarda [23,112].
  - In centers with high laparoscopic and robotic experience, a posterior minimally invasive approach might be the best recommendation for a patient with an inguinal hernia.
- A center of excellence should have all types of repairs available, not necessarily carried out by the same surgeon.
- All current strategies should NOT be viewed as competing, but as COMPLEMENTARY techniques.
- Surgeons offering advice to patients should be familiar with each approach, possible complications, and system-based practices, such that a tailored approach for inguinal hernia repair can be offered to patients.
- The present report shows that even with higher costs and operative room times, the robotic approach to inguinal hernia repair is likely to continue to evolve and become more prevalent in centers with robotic experience.

Not offering patients all forms of repair, including WW, is unfair to our patients and might be too paternalistic. It is important for all surgeons to be able to provide patients with sufficient information regarding the risks and benefits of each approach for them to make appropriate choices. This includes recommendations for WW for patients who might not be ready for an operation. The risks and benefits of other approaches, including OR times and cost, should be disclosed to patients within the context of the experience of the surgeon, center, and system practices. Currently, there is no question that the cost of RHR is higher than any other platform. It would be important to discuss this with our patients in an era of escalating health care expenditure.

With this in mind, the current reasons for carrying out each approach remain important.

#### 4.1.1. Why the OPEN Approach?

1. Reproducibility of the Lichtenstein technique with acceptable complications;
2. Low cost;
3. Ability to use regional or local anesthesia readily;
4. The recommended approach for a recurrence where the index operation was via the posterior approach.

#### 4.1.2. Why Laparo-Endoscopic Approach?

1. Gold standard for anterior recurrences and bilateral inguinal hernias.
2. Best ability to visualize the myopectinate orifice.
3. Patient-driven desire to have a laparoendoscopic approach.
4. Academic surgery centers driving the use of laparoendoscopic techniques.

#### 4.1.3. Why the Robotic Approach?

1. RHR provides a foundation for more advanced robotic cases as this provides training for faculty and residents with an operation with low rate of complications.
2. Market-driven reasons. Patients might request RHR, and this might be driving the need for surgeons and centers to be trained in providing this service.
3. Centers of excellence: centers of excellence for hernia surgery today should provide all strategies, perhaps not performed by the same surgeon, but by the group in the center.
4. Surgeons must keep up to date with technological advancements. It is possible that further advancements might be able to identify all the issues related to current problems with inguinodynia and recurrence better than surgical judgment of an individual.
5. The learning curve is substantially lower than LHR (~12–14 cases).
6. Surgeon’s comfort.

### 4.2. Limitations of the Study

There are several limitations to this study. The most substantial limitation emanates from extracting data in a retrospective fashion from all papers presented. The data that were examined in each manuscript contained highly heterogenous variables. The reporting of outcomes and objectives was extremely diverse. Because we extracted several robotic series, there was a high likelihood of bias by the investigators advocating for minimally invasive techniques. Similarly, authors focusing on cost might have been biased in extracting any potential incident with the robotic approach that might lead to a higher cost. Reporting of outcomes and complications has been a problematic issue in hernia surgery overall. This was highly visible in this report. The types of studies ranged from case reports to systematic analyses with meta-analyses. However, because this is a perspective paper, we wanted to be inclusive, such that the best viewpoint could be provided. Even with such inclusiveness, some important papers might have escaped our review. There were reports that included only a few patients, a single surgeon, and a single center, such that the pertinence of the results from these manuscripts might not be applicable to other surgeons, patients, or institutions. Similarly, there were some hernia databases that were not entirely scrutinized, such as the ones originating in Germany, as this would have made the manuscript prohibitively long. However, this represents the most comprehensive analysis on this subject today.

## 5. Conclusions

The management of GHs in the 21st century has drastically changed the practice of general surgery. It might be impossible for a surgeon to be a master of all current techniques, but a center should offer all. Surgeons should be informed of risks and benefits including the cost of all techniques to appropriately direct patients in a shared decision-model. The robotic platform has emerged as an unequivocal option for GH management. While OHR remains the technique of choice for local/regional anesthesia, posterior recurrences, and in centers that lack other platforms, centers of excellent should offer all techniques of repair as well as WW.

## Figures and Tables

**Table 1:** Result of the literature review. Comparisons are shown if they were present in the manuscript reviewed. Only comparisons of OHR vs. RHR, LHR vs. RHR, or OHR vs. LHR vs. RHR were included. Direct comparisons between OHR and LHR were excluded. OR = operative room; BHIR = bilateral inguinal hernia repair; mo = months; pts. = patients; SBO = small bowel obstruction; yr. = year; LOS = hospital length of stay; PSM = propensity score match analysis; MIS = minimally invasive; VA = Veterans Affairs; ACS = American College of Surgeons NSQIP = National; Surgical Quality Improvement Program; VASQIP = Veterans Affairs Surgical Quality Improvement Program; QoL = Quality of life; EBL = estimated blood loss.

Author (Year) [Reference]	Period of Study	Study Type	Number of Patients OHR/LHR/RHR	Main Findings
Bittner (2018) [56]	2018	Retrospective, institution study, with PSM analysis	85/83/85	No significant difference in patient satisfaction based on approach. There is a potential benefit in perception of pain by MIS techniques. One of the first PSM analyses.
Charles (2018) [30]	2012–2014	ACS NSQIP database	210/241/69	OR time was higher with RHR as well as infections and cost. Other outcomes were similar. This study is one of the first interrogating a large database.
Aiolfi (2019) [96]	2019	Systematic Review and meta-analysis = 16 studies	18,118/32,600/1500	OHR, LHR, RHR are comparable in the short term. Postoperative seroma, chronic pain, and recurrence were similar.
Huerta (2019) [17]	2005–2017	Single center case series at a VA Hospital	1100/128/71	Even in expert hands RHR is worse than LHR and OHR. Recurrence and chronic pain were higher with the LHR and RHR compared to OHR.
Pokala (2019) [83]	2013–2017	Vizient clinical database	2413/540/594	RHR had the least overall incidence of complications. OHR had the highest postoperative infection rate and longer length of stay when compared to laparoscopic and robotic approaches. RHR was more expensive than LHR. Opiate use was higher in the OHR.
Sheldon (2019) [70]	2016–2018	Retrospective institutional study	90/34/49	This paper only evaluates pain and there was no difference in repeated prescriptions in any group. There was no difference in pain requirements.
Janjua (2020) [82]	2009–2015	The Healthcare Cost and Utilization Project-State Inpatient databases and the American Hospital Association Annual Health Survey Databases	27,776/7104/1516	Robotic costs were 38% greater than those of the open and laparoscopic subsets. RHR and LHR have shorter LOS compared to OHR.
LeBlanc (2019) [77]	2016–2018	Ongoing, multicenter, comparative, open-label analysis of clinical and patient reported outcomes from RHR vs. OHR and RHR vs. LHR.	190/155/159	Fewer prescriptions for pain were better for RHR. Time to normal activity was better with MIS compared to open approach. OR times were longer for RHR vs. LHR. OR times were also longer for RHR vs. OHR in BHR.
Holleran (2021) [7]	2008–2019	VASQIP analysis	100,880/18,035/6063	Increase in LIH and RIH from 0.24% to 19.6% with decreased complications from 20.8% to 3.5% between 2008 and 2019.
Kakiashvili (2021) [66]	2014–2016	Single institution, single surgeon study.	97/16/24	OR Time RHR > LHR > OHR. OHR had greater hospital LOS. Pain was worse for OHR vs. LHR vs. RHR.
Qabbani (2021) [93]	2000–2020	Systematic review and meta-analysis: 19 studies	4280/1495/2521	RHR had longer OR time and lower re-admission rate. No other differences in outcomes. RHR complication = 10.1%, recurrence 1.2%. RHR vs. LHR longer OR time, but less complications. OHR vs. RHR longer OR time with RHR and less re-admission rate. RHR remains inferior to OHR. from (3% to 1.5%) and costs also decreased.
Tatarian (2021) [86]	2010–2016	The New York Statewide Planning and Research Cooperative System (SPARCS) administrative database	117,603/35,565/559	Only unilateral hernias were included. Overall complications, and 30 d readmission rate RHR were associated with comparable to better outcomes compared to both OHR and LHR on PSM analysis.
Varvoglis (2022) [87]	To 2021	The Abdominal Core Health Quality Collaborative database with PSM analysis	297/285/266	Surgical site infections, recurrence, requiring surgical intervention. The immediate pain scores after the operation were better for LHR vs. OHR and LHR, but QoL was equivalent one year after the operation.
Hsu (2023) [63]	2016–2019	Single institution with eight surgeons	194/305/207	This is a three-arm study of unilateral, non-recurrent IHRs. There were no differences in complications. OHR and RHR OR time were longer. Cost RHR > LHR > OHR. Recurrence OHR = 2, LHR = 0, RHR = 2. Inguinodynia was similar. More sermons in RHR. One bowel injury in LHR, one vascular in RHR.
Read (2023) [84]	2015–2020	The Florida Agency for Health Care Administration database including 86 hospitals.	19,364/12,322/4707	Cost was higher for RHR. This cost did not plateau over time but increased.
Lorenz (2024) [50]	2007–2022	Retrospective data collected on prospective single-institutional study with PSM analysis.	140/140/140	OHR patients had more comorbidities and more recurrent hernias. There was no difference in overall complications or re-admission rate. Pain was similar. RHR was more expensive > LHR > OHR. Recurrence: 3.6%, 07%, 0.7%.

## Data Availability

All data presented in this manuscript is available in the literature.
